# Assessment of a Leadership Enhancement Program for Nursing Managers of An Acute General Hospital in Hong Kong

**DOI:** 10.2174/1874434601812010133

**Published:** 2018-06-29

**Authors:** Leung Andrew Luk

**Affiliations:** Alice Ho Miu Ling Nethersole Charity Foundation, Chuen on Road, Tai Po, N. T. Hong Kong

**Keywords:** Servant leadership, Workplace well-being, Nursing job satisfaction, Pre and posttest, Reflective essay

## Abstract

**Background:**

With the aim to enhance the senior nursing managers to be caring leaders so that they can nurture their team members to be holistic care providers, a one year program has been developed with emphasis on self-reflection and self-cultivation.

**Purpose:**

It aims to evaluate the effectiveness of a one-year leadership enhancement program in an acute general hospital.

**Design & Methods:**

Both quantitative and qualitative approaches were adopted. A pre and post questionnaire survey and the content analysis of self-reflective essays were conducted.

**Results:**

The overall means of the servant leadership scale and the workplace wellness scale were significantly higher after the program. Both scales were also found to have a significantly medium level of positive correlation. Reflective essays showed positive feedbacks complementing the quantitative data that the program was well received and effective.

**Conclusion:**

A servant leadership approach may be one way for hospital management to enhance a caring environment and a more quality workforce.

## 1 INTRODUCTION

An acute general hospital with appropriate 1400 in-patient beds which is built on a strong heritage of Christian love with compassionate care has been established in Hong Kong since 1973. Hospital predecessors have already set up a good foundation in terms of structure, policy, educational activities and role models. However, compassionate care has to be cultivated by everyone in the hospital and passed from one generation to the next. The nursing administrative department believed that senior nursing managers are an important part of the staff who can uphold the caring heritage and influence their teammates. In return, junior staff will provide a high quality of care to clients and learn to be caring leaders in future. Furthermore, the nursing shortage is a common phenomenon in many advanced cities, Hong Kong is of no exception. To ensure a quality nursing workforce in a time of labor shortage, nursing managers in executing strong nursing leadership play an important role in supervising and supporting the frontline nurses [1].

With the aim to enhance the senior nursing managers to be caring leaders so that they can nurture their team members to be holistic care providers, a one year program has been developed with an emphasis on self-reflection and self-cultivation. With the belief that lives influencing lives, the program expects participants to share, to learn from one another and build up mutual support in the nursing team. The program was started in April 2016 and ended in March 2017. The contents of the program consisted of one seminar per month for 5 months. Each seminar was a 1.5-hour talk during lunch break. Then, it followed with five sessions of small group sharing with 1.5 hours during lunch break or in the afternoon. The last part of the program was a one day retreat. The total hours spent on the program were 21. This paper reports the outcomes after the implementation of this leadership enhancement program.

## 2 THE LEADERSHIP MODEL AND CONTENT OF THE PROGRAM

A number of studies show that the nurse managers and her engagement with her staff is a key factor in nurse job satisfaction despite working in a stressful environment. Satisfaction usually comes from managerial care, support and respect [2-5]. There are different management models or theories which help managers better lead and support their followers. As a hospital with strong Christian background, Jesus Christ has long be a model of leadership. He demonstrated his leadership in both his words and deeds. He claimed to come to the world to serve but not to be served [6]. He humbly washed his disciples’ feet [7]. In management, the theory “the servant as leader” proposed by Greenleaf [8] matched the leadership model shown in the bible. The servant leadership model gained a lot of evidences of its effectiveness not only in business sector, but also in the healthcare settings [9-12]. The basic idea of servant leadership is that servant leader recognizes his or her moral responsibility not only to the success of the organization but also to his or her subordinates, the organization’s customers, and other organizational stakeholders [8]. Hale and Fields [13] defined servant leadership as “ an understanding and practice of leadership that places the good of those led over the self-interest of the leader, emphasizing leader behaviors that focus on follower development, and de-emphasizing glorification of the leader” (p. 397). Beyond leading with altruism, Spears [14] identified 10 characteristics common among servant leaders: listening, empathy, healing, awareness, persuasion, conceptualization, foresight, stewardship, growth, and building community. Though servant leadership overlaps with other theories such as transformational, ethical, authentic and spiritual leadership and exhibits some behaviors as role modeling, inspirational communication, and altruism [15], it is suggested by Walumbwa, Hartnell and Oke [12] that servant leadership is theoretically distinct in several important ways. First, it includes a moral component. Second, it is uniquely concerned with the success of all organizational stakeholders. Third, servant leader acts in the best interest of the followers. Fourth, servant leader engages in self-reflection to reduce leaders’ arrogance.

The servant leadership model is adopted in this program to encourage participants to put subordinates first, to be humble and learn from one and other. On the other hand, it is also important to take care of oneself before taking care of others [16]. A personal wellness, a holistic view of a person including physical, psychosocial and spiritual aspects is also introduced. Participants help to understand oneself holistically and develop more in their potentials to be a servant leader at the same time a holistic person to care for oneself and others from a holistic perspective. Details of the program can be seen in (Table **1**).

## 3 METHOD

Both quantitative and qualitative approaches were adopted in the assessment of the effectiveness of the program. A pre and post questionnaires were sent to participants of the program. After the completion of the program, participants were also asked to return a self-reflective essay for content analysis. Invitations were sent to all senior nursing managers of the hospital including Department Operation Managers (DOM), Nursing Consultants (NC), Senior Nursing Officers (SNO), Ward Managers (WM) and Advanced Practice Nurses(APN) of all units. Totally 42 senior nursing managers joined the program. All participants joined the program voluntarily. They are free to fill in the pre and post tests of the survey and submit with the anonymity of questionnaire which was only marked by self- assigned code.

### 3.1 Instruments

The questionnaire consists of two sets of scales. The first one is a general measure of servant leadership (Appendix **A1**) which composes of 7 categories: forming relationships with subordinates, empowering subordinates, helping subordinates grow and succeed, behaving ethically, having conceptual skills, putting subordinates first, and creating value for those outside of the organization [17]. Each category contains 2 items with the complete set of 14 items. Participants rated each of the items on a 5-point scale from 1=to a very small extent to 5= to a great extent. The alpha reliability of the overall scale was 0.98. Participants were asked to rate themselves as servant leaders who lead their followers in their service units.

The second set is a measure of well-being in the workplace (Appendix **A2**) which composes of 4 domains: work satisfaction, organizational respect for the employee, employer care and intrusion of work into private life. This Workplace Well-being Questionnaire (WWQ) contains 31 items. The participants were asked to rate the items that best represented their current and most relevant work situation on a 5-point scale: 0, not at all; 1, slightly; 2, moderately; 3, very; and 4, extremely true. This questionnaire was found to have a high test-retest reliability, Pearson r=0.91 for the overall scale [18] and a good validity [19].

## 4 Results

### 4.1 Pre and Post Questionnaire

Totally 42 senior managers have taken part in the program. Twenty-two were DOM/NC/SNO, 20 were WM/APN. Ten are male and 32 female. Age was within 35 to 55. The average attendances of the seminars of DOM group and WM group were 72% and 83%, respectively. The average attendances of the small groups of DOM group and WM group were 51% and 61%, respectively. The total average attendances of all participants were 67%.

Out of 42, 26 participants have returned their pre and post-test questionnaires, the returned rate was 62%. Regarding the scoring of the servant leadership scale, participants got scoring from 4 out of 7 categories and the overall mean significantly higher after the program. Details can be seen in (Table **2**).

Relating to the workplace wellness scale, participants got scoring from 3 out of 4 domains and the overall means significantly higher after the program. Details can be seen in Table **3**.

When using the Pearson correlation test for the outcomes of leadership scores and workplace wellness score, a medium level of positive correlation was found between these variables with statistically significant differences r=0.427, p=<0.05. Details can be seen in Table **4**.

### 4.2 Reflective Essays

There were totally 7 essays received. Low submission rate may be due to managers’ busy schedule and lack of the habit of writing essays. However, all returned essays provided rich data for content analysis. The content of their reflection was analyzed into 2 main areas: the content of the program and effects on Participants.

#### 4.2.1 Content of the Program

Words relating to the content of the program such as well organized, inspiring, enjoyable, fruitful, and meaningful can be found. Participants also showed appreciation to the course organizer, speakers and group facilitator. Some of their sharing are shown as below.

#### 4.2.2 Well Organized, Inspiring

This is a pretty good course with comprehensive content and thoughtful arrangement. The sharing from different lecturers and leaders gave me many brainstorming and stimulate me to have a deeper understanding of the key elements of being a leader. However, I gained much more from the sharing in the tutorial group. I felt grateful that this course just right in time to guide my direction and my way. I could not stay in the past, I was determined to have a breakthrough and make a change. (Essay 7)

#### 4.2.3 Enjoyable, Enlightening

I really enjoy this program and appreciate the effort from the coordinators. In this program, I could realize the different aspects of myself. This program was full of touching, sharing and stories and gave me a chance in a tranquil environment to revisit the value, mission and my dream in nursing. The topics of this program were diversified and the expert speakers provided the substantial content to enlighten me. The program made me think my work, family and life in multi-dimensions. (Essay 1)

#### 4.2.4 Fruitful

To me, the course is a bottle of spiritual nutrients throughout my year in 2016-17. Having worked in nursing for many years, there are times that I have lost direction, shifted/lost the meaning and value of nursing or even in life because of all the chaos. Nonetheless, all the sessions have energized my spirit, to refresh and keep going. Finally, I would like to thank the course coordinators for their dedication and effort for the future nursing leaders. (Essay 4)

#### 4.2.5 Effects on Participants

Three themes emerged with more than half of them mentioned in their sharing: Reflecting and refreshing; gain more self-understanding and reviving. Some of their sharing are illustrated below.

#### 4.2.6 Reflecting and Refreshing

I think I am lucky to be one of the members of the program that I could have opportunities and time to stop and think. In fact, I can do it while I am free, but usually, I will be distracted by other staff. Honestly, I seldom ‘stop and think’ in my day-by-day busy working life which drains all my energy at the end of the working day. I think it is good for me to have an ‘official’ time to think more about myself, my career and my future. (Essay 6)

I was wrongly assumed that I have already mastered many techniques at my age getting to retired and thought that this was merely a day camp with nothing new and time could easily be passed. Unexpectedly, the first item “technique cards” already made me rethink that I turned out haven’t made good use of many techniques.

In the afternoon session, Dr. Choi was invited to be a speaker and I gained a lot from his sharing. He made me think again how to maintain my holistic health. Every day is a busy day, it does not only make you feel tired physically but also spiritually. We really need to take care of our holistic health in order to take care of the people around us. (Essay 5)

#### 4.2.7 Gain More Self-Understanding

Although I thought the tutorial group would not be as attractive as a lecture, however, it gave me a chance to explore myself. I came to understand more of my strengths and weaknesses, and the type of “leader” I belong to. It acts like a mirror to allow me clearly see how could I live my life and what could I contribute to nursing. After recovery from illness, each day is a bonus for me. I really did not want to waste my life. However, in the busy days, I am getting lost…. (Essay 7)

The exercises in sharing sessions can enhance self-understanding and know others’ strengths. The group with similar seniority from a different background can create non-political resonance. By sharing with one and other, we can know others’ perspectives and find the way out. Greater exposure can cultivate support and networking with each other to be companions on the lonely road. (Essay 2)

#### 4.2.8 Reviving

PPEP program is a booster on my way. It refreshes my energy to continue. Besides, to sustain the seeding on me, I shall try my best to nourish those I meet. I believe little thing can make differences. Life influences live. (Essay 2)

The fire in my heart being rekindled. Nowadays, I would not focus on how much was given to me by others, but how much I can do for others. In the past, I was being nurtured by my seniors. Today, I hope to be a blessing for others, nurture the juniors and help them to grow. Hoping that my hospital has more and more blessings in the future. (Essay 7)

The focus of this day camp was not on teaching techniques, but have reflections on life and work. We reflected and discussed our works through the movie “The Five People You Meet in Heaven”. My conclusion is, I have to change my attitude, to regain my original intention in nursing, to rekindle my passion, and to love my work. (Essay 5)

## 5 DISCUSSION

The pre and post-tests showed that there was an overall improvement in servant leadership particularly in the areas of empowering subordinates, behaving ethically, having conceptual skills and creating values for those outside of the organization. There may be several possible reasons for this positive result. First, the nursing administration demonstrated the respect and care to senior managers in organizing this program with the aim to enhance their leadership knowledge and skill. This may exert a positive influence on them to act in the same way to their followers. Second, this program introduced the philosophy and practice of the servant leadership and encourage managers to act in the best interest of their followers. More leadership potentials are unleashed and participants may also influence one another. Third, during the whole year of implementation, a culture to serve and care is much more reinforced, which help participants in the program to put theory into practice.

The pre and post tests also showed that there was an improvement in the workplace wellbeing in all areas and overall mean except in the area of “intrusion of work in private life”. It is understandable that participants will perceive more respect from the organization and employer care since the nursing administration has invited training experts in designing and facilitating the whole program for them. The program coordinator sat in the whole program for support. Regarding no improvement in the domain of “intrusion of work in private life”, some possible reasons may be: first, in time of shortage of nursing workforce, overtime is not uncommon for nursing management in nearly all acute hospitals in Hong Kong. Furthermore, the studied hospital serves a densely populated region with more than 500,000 population. It is sometimes difficult not to bring paperwork back to home or staying late to finish work at hand. Finally, maintaining a work/life balance takes time for improvement.

The correlation test showing a medium level of the positive relation of servant leadership with workplace wellbeing provides a further support of the effectiveness of servant leadership which is also consistent with other studies overseas [9, 20, 21]. It is worth promoting this type of program to other hospitals so that senior nursing managers may have a better support and a healthier work life. In turn, nursing staff and patients are supported and cared for. This type of leadership is also supported by a leadership model which is more appropriate to healthcare setting and aligned with compassionate leadership. In a compassionate health care setting, patients and staff would feel listened to, supported, and cared for [22].

With regard to the reflective essays, though not many participants submit theirs, the feedbacks compliment the quantitative data that the program is well organized and effective. It provides time and opportunities for senior nursing managers for reflection and refreshing. The serving hearts are revived. They are re-motivated to serve and pass the compassionate care to their followers.

## 6 LIMITATIONS

Regarding the quantitative approach, it is only a pre and post test designs and the long-term effect of the servant leadership is not known. If assessment after six and 12 months can be taken, a better picture may be obtained. Furthermore, this study only provides one perspective from the senior managers, if views from their followers and patients can be considered, a more comprehensive picture can be seen. Relating to the qualitative approach, if focus group or individual interview is conducted, more in-depth data can be collected for content analysis.

### 6.1 Implications for Practice and Research

Moving towards the 21^st^ century, there will be a great demand for hospital services due to increasing the aging population in Hong Kong. A labor shortage with possible declining work satisfaction will be the challenges of hospital administrators. A servant leadership approach is shown to be one way for hospital management to enhance a caring environment and a more qualified workforce. This program based on a servant leadership model, on one hand, helps nurse managers to lead by follower-center than self-center and use of self-reflection and self-cultivation. On the other hand, it also reminds them that a leader is no more than a human who needs to take care of oneself while taking care of others. Since it is an initial project of the hospital for training of nurse managers, further research should be conducted to assess its effectiveness for training another batch of nurse managers on a more solid mixed research approach.

## CONCLUSION

A leadership enhancement program based on a servant leadership model with emphasis on self-reflection and self-cultivation was found to be effective in promoting nurse leaders well-being in one acute general hospital. This program is worth to be considered as an in-service leadership training program in other acute hospitals. However, further research should be conducted at the same time to assess its effectiveness to build up more evidence.

## Figures and Tables

**Table 1:** The details of the Personal and Professional Enhancement Program (PPEP).

Seminar	Topics
	**How to influence others as a leader: Practical experiences** **Objectives:** - share own experiences in learning to be a leader - encourage a sharing and supporting culture
	**Enhancing your EQ at work** **Objectives: -** understand the most frequently expressed emotions - learn how to manage negative emotions in a constructive way
	**Self-care and resilience in nursing** **Objectives: -** identify stressors and risks at work - learn how to build up one’s resilience in adversity
	**Holistic care – to care with soul** **Objectives:** - reflect self as a caring agent in a holistic view - learn how to regain a work / life balance
	**Book sharing** **Objectives:** - introduce different types of books relating to leadership - encourage reading as one important way for self-cultivation
**Group** **Sharing**	**Topics**
	**Self-cultivation - personal wellness** **Content:** - take self-assessment on personal wellness - share ways for maintaining personal wellness
	**Self-cultivation – An exploration of Self relating to leadership qualities** **Content: -** take self-assessment on personal personality - share how one’s personality affecting the leadership style
	**Self-cultivation – A servant leadership model as a holistic approach** **Content: -** revisit the characteristics of servant leadership - take self-assessment on servant leadership
	**Management case sharing - staff** **Content:** - share different approaches in managing difficult staff - identify ways of integration of theory into practices
	**Self-cultivation – Understanding Self leadership** **Content: -** take self-assessment on self-leadership - sharing ways of enhancing self -leadership
**Retreat**	**Topics**
	**Objectives: -** consolidate previous learnt concepts into clinical application - enrich personal strength with holistic components **Content:** - Movie appreciation for life reflection - Spiritual guidance on professional enhancement

(*p <.05, **p<.001)

**Table 2:** Servant leadership: Paired Samples t-test results.

-		Mean (SD)	N	t	df	Sig. (2-tailed)
Forming relationships with subordinates	pre-test	3.60 (.51)	26	-1.127	25	0.271
	post-test	3.73 (.59)	26			
Empowering subordinates	pre-test	3.63 (.50)	26	-2.065	25	.049*
	post-test	3.87 (.58)	26			
Helping subordinates grow and succeed	pre-test	3.44 (.45)	26	-1.397	25	0.175
	post-test	3.62 (.62)	26			
Behaving ethically	pre-test	3.96 (.42)	26	-2.301	25	.030*
	post-test	4.15 (.39)	26			
Having conceptual skills	pre-test	3.81 (.43)	26	-2.388	25	.025*
	post-test	4.06 (.36)	26			
Putting subordinates first	pre-test	3.90 (.49)	26	-0.926	25	0.363
	post-test	4.00 (.53)	26			
Creating values for those outside of organization	pre-test	2.92 (.91)	26	-3.923	25	.001*
	post-test	3.52 (.77)	26			
Overall mean	pre-test	3.61 (.30)	26	-4.027	25	.000**
	post-test	3.85 (.38)	26			

(*p <.05, **p<.001)

**Table 3:** Work wellbeing: Paired Samples t-test results.

-		Mean (SD)	N	t	df	Sig. (2-tailed)
Work satisfaction	pre-test	2.72 (.42)	26	-3.387	25	.002*
	post-test	3.02 (.39)	26			
Organizational respect for the employee	pre-test	2.46 (.45)	26	-3.277	25	.003*
	post-test	2.77 (.37)	26			
Employer care	pre-test	2.43 (.61)	26	-3.062	25	.005*
	post-test	2.77 (.47)	26			
Intrusion of work into private life	pre-test	1.81 (.67)	26	-1.076	25	.292
	post-test	1.90 (.66)	26			
Overall mean	pre-test	2.48 (.37)	26	-3.757	25	.001*
	post-test	2.70 (.29)	26			

(*p <.05)

(Items of factor 4 were reverse scored when calculating the overall mean.

Higher overall score indicates better work wellbeing)

**Table 4:** Correlation between pre-test and post-test means of work well-being and servant leadership.

-		Servant leadership *pre-test*	Servant leadership *post-test*
Work well-being *pre-test*	Pearson Correlation	-.040	-.071
	Sig. (2-tailed)	.845	.730
Work well-being *post-test*	Pearson Correlation	.104	.427*
	Sig. (2-tailed)	.612	.030

*Correlation is significant at the 0.05 level (2-tailed)

**Table A1:** 

	-	To a *Very Small* Extent				To a *Great* Extent
		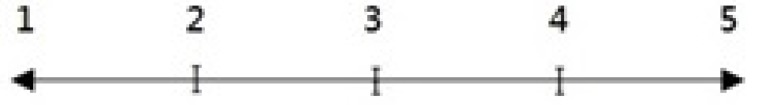				
1	I spend time to form quality relationships with my subordinates.	1	2	3	4	5
2	I create a sense of community among my subordinates.	1	2	3	4	5
3	My decisions are influenced by my subordinates’ input.	1	2	3	4	5
4	I try to reach consensus among my subordinates on important decisions.	1	2	3	4	5
5	I am sensitive to my subordinates’ responsibilities outside the work place.	1	2	3	4	5
6	I make the personal development of my subordinates a priority.	1	2	3	4	5
7	I hold my subordinates to high ethical standards.	1	2	3	4	5
8	I do what I promise to do.	1	2	3	4	5
9	I balance concern for day-to-day details with projections for the future.	1	2	3	4	5
10	I display wide-ranging knowledge and interests in finding solutions to work problems.	1	2	3	4	5
11	I make my subordinates feel like they work with me, not for me.	1	2	3	4	5
12	I work hard at finding ways to help others be the best they can be.	1	2	3	4	5
13	I encourage my subordinates to be involved in community service and volunteer activities outside of work.	1	2	3	4	5
14	I emphasize the importance of giving back to the community.	1	2	3	4	5

**Table:** Table A2

-		Not at all	Slightly	Moderately	Very	Extremely true
1	Is your work fulfilling?	0	1	2	3	4
2	Do your daily work activities give you a sense of direction and meaning?	0	1	2	3	4
3	Does your work bring a sense of satisfaction?	0	1	2	3	4
4	Does your work increase your sense of self-worth?	0	1	2	3	4
5	Does your job allow you to re-craft your job to suit your strengths?	0	1	2	3	4
6	Does your work make you feel that, as a person, you are flourishing?	0	1	2	3	4
7	Do you feel capable and effective in your work on a day-to-day basis?	0	1	2	3	4
8	Does your work offer challenges to advance your skills?	0	1	2	3	4
9	Do you feel you have some level of independence at work?	0	1	2	3	4
10	Do you feel personally connected to your organization’s values?	0	1	2	3	4
11	In general terms, do you trust the senior people in your organization?	0	1	2	3	4
12	Do you believe in the principles by which your organization operates?	0	1	2	3	4
13	Do you feel content with the way your organization treats its employees?	0	1	2	3	4
14	Do you feel that your organization respects the staff?	0	1	2	3	4
15	How satisfied are you with your organization’s value system?	0	1	2	3	4
16	Compared with your organization’s “ideal values,” to what degree are actual work values positive?	0	1	2	3	4
17	Do people at your work believe in the worth of the organization?	0	1	2	3	4
18	At a difficult time, would your boss be willing to lend an ear?	0	1	2	3	4
19	Is your boss caring?	0	1	2	3	4
20	Do you feel that your boss is empathic and understanding about your work concerns?	0	1	2	3	4
21	Does your boss treat you as you would like to be treated?	0	1	2	3	4
22	Does your boss shoulder some of your worries about work?	0	1	2	3	4
23	Do you feel your transactions with your boss are, in general, positive?	0	1	2	3	4
24	Do you believe that your employer cares about his or her staff‘s well-being?	0	1	2	3	4
25	Does your work eat into your private life?	0	1	2	3	4
26	Do you feel stressed in organizing your work time to meet demands?	0	1	2	3	4
27	Do you feel excessively pressured at work to meet targets?	0	1	2	3	4
28	After work, do you find it hard to wind down?	0	1	2	3	4
29	Do you find yourself thinking negatively about work outside of work hours?	0	1	2	3	4
30	Do you feel that you can separate yourself easily from your work when you leave for the day?	0	1	2	3	4
31	Does your work impact negatively on your self-esteem?	0	1	2	3	4

## APPENDIX 1

The statements listed below describe the leadership style of a leader. Please read each statement. Circle the number to the right of each statement that best describes you.

## APPENDIX 2

Choose the option that best represents your current and most relevant work situation (*i.e.*, the work role where you spend most of your time and with who you have most contact) by circling the appropriate number.
